# Case Report: A rare case of Pneumocystis jirovecii infection with left hydropneumothorax following immunotherapy for stage IVB clear cell renal cell carcinoma

**DOI:** 10.3389/fmed.2026.1784855

**Published:** 2026-03-23

**Authors:** Li Jiang, Tao Ye, Haibo Cai, Fan He

**Affiliations:** 1 Guizhou University of Traditional Chinese Medicine, Guiyang, Guizhou, China; 2 First Affiliated Hospital of Guizhou University of Traditional Chinese Medicine, Guiyang, Guizhou, China

**Keywords:** hydropneumothorax, immune checkpoint inhibitors, immunotherapy, PJP, renal carcinoma

## Abstract

**Background:**

Pneumocystis jirovecii pneumonia (PJP) is an opportunistic infection that predominantly affects immunocompromised individuals, most commonly HIV-infected patients with significantly reduced CD4+lymphocyte counts, and is associated with high clinical mortality. Currently, there are few reports of pneumothorax secondary to PJP, and most cases occur in HIV-infected populations. However, PJP complicated by hydropneumothorax in cancer patients receiving immunotherapy is exceedingly rare, with limited reports in the literature. To our knowledge, this article reports a rare clinical case of Pneumocystis jirovecii infection complicated by left-sided hydropneumothorax in a patient with stage IVB clear cell renal cell carcinoma after immunotherapy, aiming to provide valuable insights for the early diagnosis and management of PJP and its complications in cancer patients undergoing immunotherapy.

**Case:**

A 57-year-old male patient had previously undergone surgical treatment for left renal clear cell carcinoma, and developed recurrent metastases to the descending colon, liver, and upper pole of the left kidney after surgery, with a clinical stage of T4NxM1 stage IVB. After receiving targeted combination immunotherapy with sequential PD-1 inhibitors (toripalimab) plus anti-angiogenic agents (sunitinib, axitinib)—a regimen that enhances anti-tumor immunity but may disrupt pulmonary immune homeostasis—the patient gradually developed progressive dyspnea, chest tightness, hypoxemia, and anuria. Multiple auxiliary examinations were performed clinically, including chest X-ray, bronchoalveolar lavage, and metagenomic sequencing of pathogenic microorganisms. Based on the above examination results, the final diagnosis was Pneumocystis jirovecii pneumonia complicated by left-sided hydropneumothorax.

**Conclusion:**

Although PJP complicated by hydropneumothorax after immunotherapy is rare, it should be considered as a possible etiology when cancer patients develop progressive dyspnea with difficulty maintaining oxygen saturation after receiving immune checkpoint inhibitor-based therapy, particularly in the context of immune checkpoint inhibitor use. While biomarkers for predicting immunotherapy efficacy and irAEs are well-studied, the identification of specific biomarkers for predicting opportunistic infections like PJP in this context remains an area of active research.

## 1 Introduction

Pneumocystis jirovecii pneumonia, also known as Pneumocystis carinii pneumonia, is a common opportunistic infection in immunocompromised individuals, particularly patients with human immunodeficiency virus/acquired immunodeficiency syndrome (HIV/AIDS). It is a major risk factor for patient mortality, and the mortality rate increases significantly in patients with concurrent pneumothorax (1, 2). With the increasing use of immunosuppressive therapy, this infection has also become more prevalent in non-HIV patients (3, 4). However, to our knowledge, PJP complicated by hydropneumothorax in cancer patients with a history of tumor immunosuppression, particularly after immune checkpoint inhibitor therapy, remains extremely rare and underreported. This article reports a case of a patient with left renal clear cell carcinoma and distant metastases who developed progressive dyspnea after receiving targeted combined immunotherapy including PD-1 inhibitor combined with sunitinib and PD-1 inhibitor combined with axitinib, and was ultimately diagnosed with Pneumocystis jirovecii pneumonia complicated by left-sided hydropneumothorax, which resulted in death.

## 2 Case

A 57-year-old male patient, with a height of 167 cm and a weight of 55 kg, was admitted to the hospital on August 6, 2025, due to “more than 1 year after surgery for left renal clear cell carcinoma, recurrence with abdominal and liver metastases for more than 7 months, and anuria for 17 h.” The patient had a history of intermittent alcohol consumption for more than 40 years and had abstained from alcohol for more than 10 months. He denied a history of smoking, HIV infection, syphilis, hepatitis B, drug abuse, or long-term drug injection.

In April 2024, the patient was diagnosed with left renal clear cell carcinoma and underwent a “partial nephrectomy.” Postoperative pathology confirmed clear cell renal cell carcinoma. In December 2024, the patient received two cycles of toripalimab (240 mg, intravenously on day 1 of each 21-day cycle) combined with sunitinib (50 mg orally once daily, 4 weeks on/2 weeks off). From February 2025 to August 2025, the regimen was switched to toripalimab (240 mg, day 1 every 21 days) plus axitinib (5 mg orally twice daily, continuous dosing). A total of 12 cycles of toripalimab plus axitinib were administered. The patient did not receive corticosteroids or any other immunosuppressive agents (e.g., mycophenolate, calcineurin inhibitors, TNF-*α* blockers) at any point before or during the illness.

**Figure 1:**
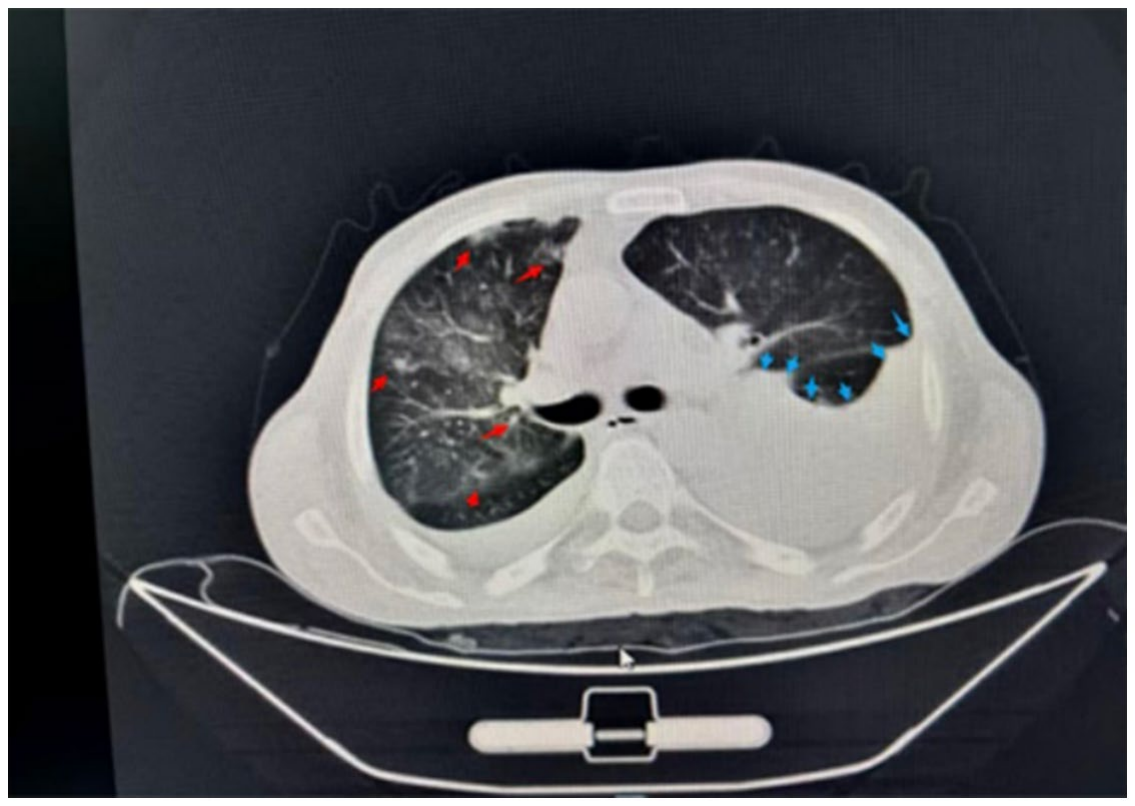
Chest CT scan on August 6, 2025, showing inflammation in the right lung (red arrow) and a large amount of left-sided pleural effusion (blue arrow).

**Figure 2:**
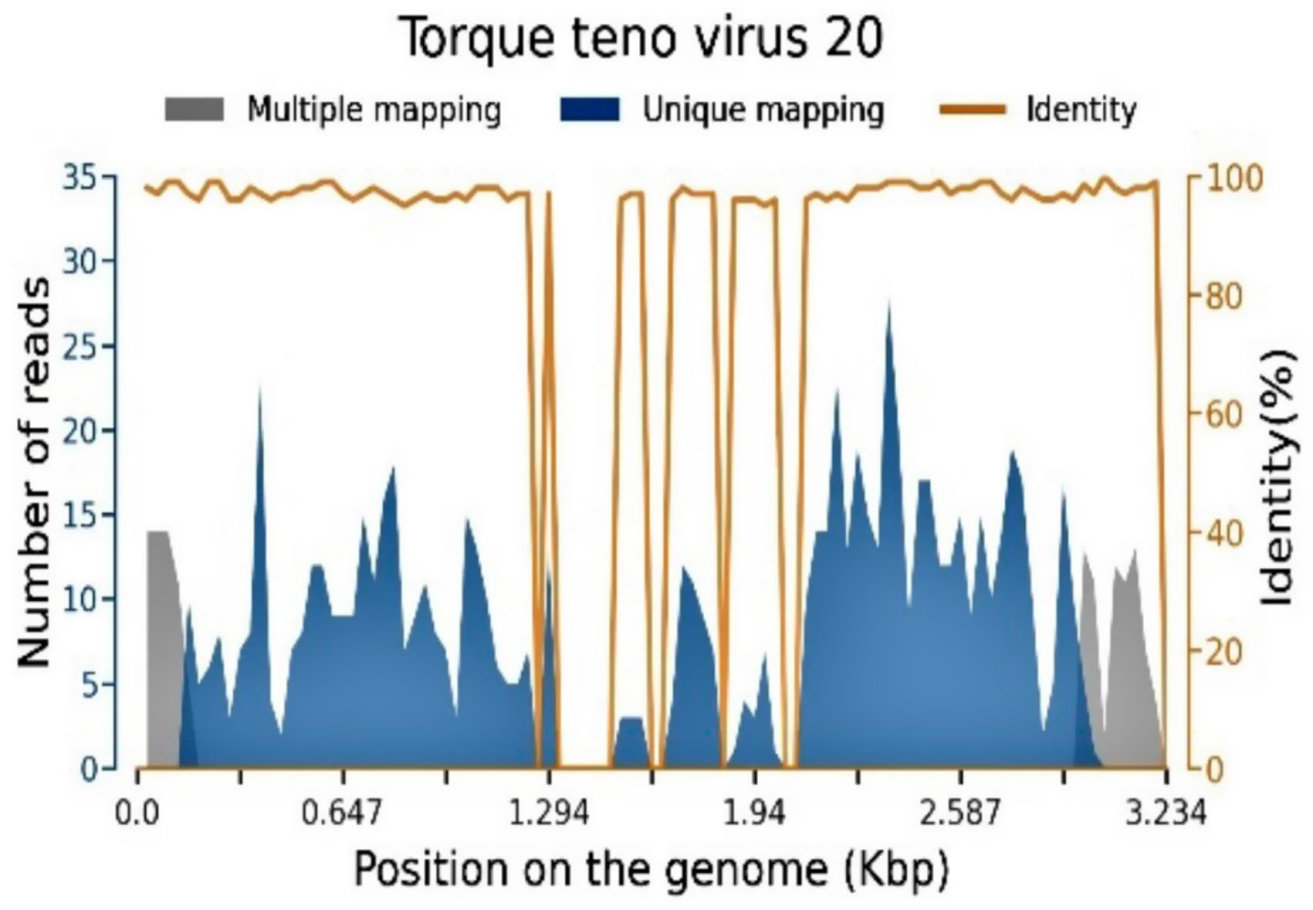
Sequence distribution map: Only species with ≥3 detected non-redundant specific sequences are shown in this section.

After immunotherapy, the patient gradually developed wheezing, shortness of breath, progressive dyspnea, and decreased blood oxygen saturation. Blood gas analysis revealed type I respiratory failure. Enhanced chest and whole abdomen CT showed bilateral pulmonary emphysema, diffuse ground-glass opacities and interstitial inflammatory infiltration in the bilateral lung parenchyma (typical CT patterns of PJP), multiple patchy consolidation inflammatory lesions in the right lung lower lobe, and bilateral pleural effusion (a large amount on the left side with obvious liquid level formation, no visible pleural thickening or adhesion) (as shown in Figure 1). The (1–3)-β-D-glucan level was 107.8000 pg/mL (clinical cutoff value: ≥80 pg./mL for positive screening, detection unit: picogram per milliliter). Laboratory tests for confirmatory diagnosis were completed simultaneously, including lactate dehydrogenase (LDH) detection with a result of 781 U/L (normal reference range: 135–225 U/L), and qPCR for Pneumocystis jirovecii was not performed due to the clinical urgency of the patient’s condition; silver stain microscopy of bronchoalveolar lavage fluid showed positive cystic structures of Pneumocystis jirovecii. On August 11, 2025, metagenomic next-generation sequencing (mNGS) of bronchoalveolar lavage fluid was performed using the Illumina NovaSeq platform with a bioinformatics analysis pipeline of BWA-MEM for sequence alignment and Kraken2 for species annotation, the positive detection threshold was set as ≥1 non-redundant specific sequence, which detected Pneumocystis jirovecii (1 non-redundant specific sequence) and cytomegalovirus (CMV) (903 non-redundant specific sequences) (as shown in Table 1 and Figure 2). The diagnosis of Pneumocystis jirovecii infection was adjudicated by the combination of clinical manifestations (progressive dyspnea, hypoxemia), positive (1–3)-β-D-glucan screening, silver stain microscopy positivity, typical PJP-related imaging features, and mNGS detection of specific pathogen sequences, despite the low mNGS signal of Pneumocystis jirovecii, which was considered to be related to the early stage of mNGS detection and the massive exudation of alveolar fluid leading to low pathogen load in the lavage fluid. The patient was treated with oral co-trimoxazole tablets (1,210 mg sulfamethoxazole + 247.5 mg trimethoprim per tablet) every 6 h for antifungal therapy.

**Table 1:** Metagenomic next-generation sequencing (mNGS) of pathogenic microorganisms in bronchoalveolar lavage fluid.

				Complex/species		
Type	Name	Sequence count	Relative abundance	Name	Sequence count	Coverage
Fungal list						
Fun	Pneumocystis	1	4.0%	*Pneumocystis jirovecii*	1	0.0006%
DNA/RNA virus list						
DNA	Alphatorquevirus	903	95.053%	*Torque teno virus 20*	774	90.1051%
List of suspected colonizing and/or background microorganisms						
G+	Cutibacterium	6	–	*Cutibacterium acnes*	5	–

**Figure 3:**
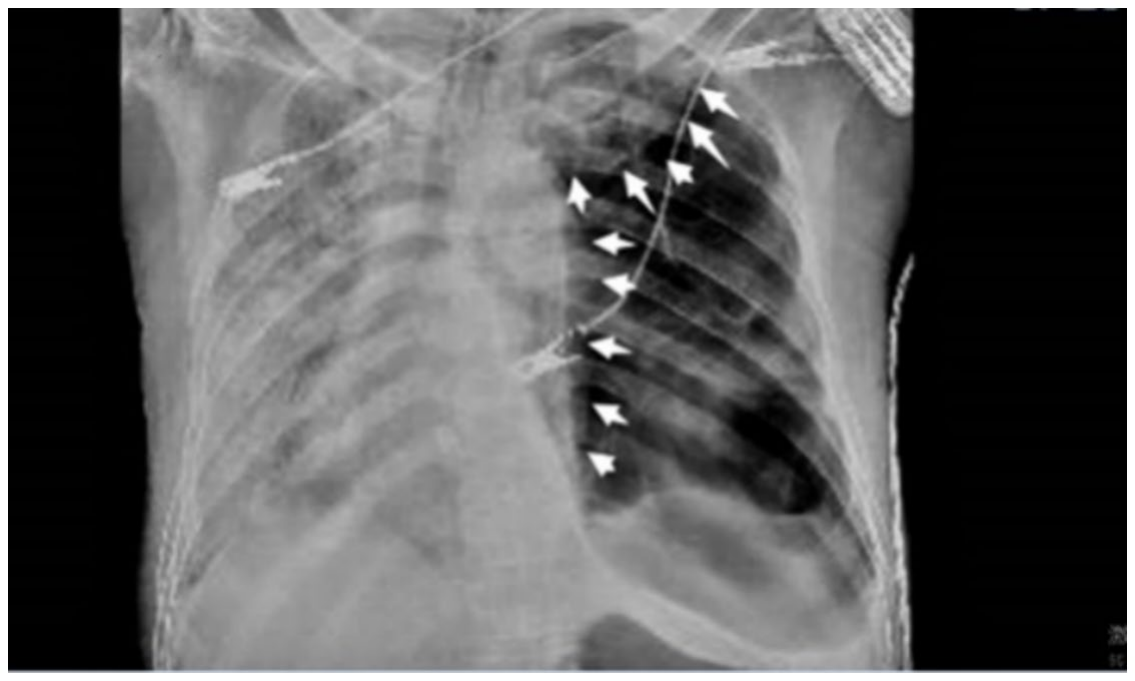
Chest radiograph on August 17, 2025, showing left-sided hydropneumothorax (white arrow) with approximately 70% compression of the left lung.

However, the patient’s condition continued to deteriorate progressively. After being transferred to the intensive care unit (ICU), the antibiotic treatment was upgraded to imipenem/cilastatin sodium to enhance anti-infective therapy, and non-invasive ventilator-assisted ventilation was used to improve oxygenation. However, hypoxemia still could not be corrected. A bedside chest radiograph showed left-sided hydropneumothorax with a clear air-liquid level in the left thoracic cavity (approximately 70% compression of the left lung parenchyma, upward displacement of the left hilum, mediastinal slight right shift), and bilateral pulmonary diffuse ground-glass opacities with patchy exudative lesions (consistent with the progressive PJP imaging features) (as shown in Figure 3).

Based on the patient’s medical history, clinical symptoms, and laboratory tests, the diagnoses were: 1. Severe pneumonia (Pneumocystis jirovecii infection); 2. Left-sided hydropneumothorax; 3. Acute pulmonary edema; 4. Type I respiratory failure; 5. Severe acute respiratory distress syndrome (ARDS); 6. Sepsis and septic shock; 7. Postoperative recurrence of left renal clear cell carcinoma with metastases to the descending colon, liver, and upper pole of the left kidney (T4NxM1 stage IVB). Despite active treatment, the patient’s respiratory failure could not be corrected, and his condition deteriorated progressively, resulting in death.

Treatment Course and Outcome. Following diagnosis, the patient received oral trimethoprim-sulfamethoxazole (TMP-SMX, 1210 mg sulfamethoxazole + 247.5 mg trimethoprim per tablet) every 6 h. Due to progressive hypoxemia, non-invasive ventilation (NIV) was initiated on August 7, 2025, but oxygenation continued to deteriorate, necessitating endotracheal intubation and invasive mechanical ventilation on August 17. A left-sided chest tube was inserted on August 17.23:00 for hydropneumothorax, with immediate drainage of 800 mL of serosanguinous fluid and air; however, a persistent air leak remained. Anuria developed on August 6; continuous renal replacement therapy (CRRT) was started on August 7 for acute kidney injury (serum creatinine 4.2 mg/dL, urea 58 mg/dL). No adjunctive corticosteroids were administered for PJP, as the patient’s hypoxemia was already severe at the time of diagnosis and there was concern for exacerbating opportunistic infections. Despite intensive support, the patient developed refractory septic shock and multi-organ failure. Death occurred on August 23, 2025, at 01:50. The immediate cause of death was determined to be severe acute respiratory distress syndrome secondary to PJP, complicated by septic shock.

## 3 Discussion

### 3.1 Mechanism of action of anti-PD-1 inhibitors and their association with PJP infection

In recent years, the incidence of PJP has gradually increased in immunocompromised populations, particularly in non-HIV-infected patients. These patients often present with more insidious onset, more rapid progression, and poorer prognosis (5). Current research suggests that inflammatory injury and immunosuppression play critical roles in the occurrence and development of PJP, and are significantly associated with the prognosis of non-HIV-infected PJP patients (6).

Anti-PD-1 inhibitors exert their anti-tumor effects by blocking the interaction between PD-1 and programmed death ligand 1 (PD-L1). PD-1 is an inhibitory receptor on the surface of T cells. When it binds to PD-L1 on tumor cells or antigen-presenting cells, it inhibits T cell activation and function, helping tumor cells evade immune surveillance. By blocking this pathway, anti-PD-1 inhibitors can release the inhibition of T cells and enhance their anti-tumor immune response. However, this enhanced immune response may trigger immune-related adverse events (irAEs), which are caused by the immune system attacking the body’s own tissues (7, 8).

The anti-tumor mechanism of anti-PD-1 inhibitors is closely related to the remodeling of the immune microenvironment, mainly reflected in the following aspects:

#### 3.1.1 Core mechanism of action of anti-PD-1 inhibitors

Enhanced T cell activation and effector function: Blockade of the PD-1/PD-L1 axis can restore T cell proliferation, cytokine secretion (such as interferon-*γ*), and cytotoxicity, thereby enhancing their ability to recognize and kill tumor cells (9–11). While enhancing anti-tumor immunity, this disruption of immune checkpoints can also lead to dysregulation of the immune system, potentially increasing susceptibility to opportunistic infections like PJP.

Immune microenvironment remodeling: Anti-PD-1 therapy can promote the infiltration of immune cells such as CD8+T cells and macrophages into tumor sites, and regulate the function of tumor-associated macrophages (TAMs) (12). The expression level of PD-1 on TAMs increases with the progression of cancer stage, and tumor immune escape is promoted by inhibiting phagocytosis. Removal of PD-L1 can enhance the phagocytic function of macrophages and reduce tumor burden (13).

Regulatory T (Treg) cell modulation: Anti-PD-1 therapy can unleash anti-tumor CD8+T cell responses, but it may also induce the expansion of immunosuppressive regulatory T cells (Tregs) through interleukin-2 (IL-2) secreted by CD8 + T cells. Combination therapy with anti-ICOSL can interfere with this process and enhance tumor control efficacy. The expansion of immunosuppressive Tregs, potentially induced by anti-PD-1 therapy, could contribute to a compromised immune response against opportunistic pathogens like Pneumocystis jirovecii, thereby increasing the risk of PJP.

#### 3.1.2 Mechanistic association between the PD-1/PD-L1 pathway and PJP infection

Regarding the specific mechanism underlying the association between anti-PD-1 inhibitors and PJP infection. Importantly, although immune checkpoint inhibitors are not classical immunosuppressive agents, they may create an immunologically vulnerable state through multiple converging factors. In this case, the patient’s risk for PJP was likely multifactorial: (1) cumulative immunotherapy with sequential PD-1 blockade and anti-angiogenic agents; (2) cancer-related cachexia and hypoalbuminemia (albumin 2.780 g/dL); (3) no prior corticosteroid exposure, but prolonged immune activation may have led to immune exhaustion; and (4) possible subclinical impairment of T cell function due to advanced-stage malignancy. These overlapping risk factors, rather than direct immunosuppression by ICIs alone, contributed to the fatal PJP event. On the one hand, PD-1 deficiency can promote macrophage activation and T helper type 1 (Th1)/T helper type 17 (Th17) responses, which is particularly significant in Pneumocystis pneumonia (14). On the other hand, PD-L1 is overexpressed in neutrophils of immunocompromised, non-HIV-infected PJP patients (15). This suggests that the PD-1/PD-L1 pathway plays an important role in maintaining the body’s immune balance against Pneumocystis. When anti-PD-1 inhibitors block this pathway, although anti-tumor immunity is enhanced, this balance may be disrupted, leading to reduced Pneumocystis clearance capacity or abnormal inflammatory responses, and ultimately increasing the risk of PJP infection (16). Case reports have confirmed that lung cancer patients can develop PJP infection after treatment with pembrolizumab (an anti-PD-1 inhibitor), which is consistent with the clinical characteristics of this patient.

### 3.2 Clinical diagnosis and therapeutic challenges of PJP

While Pneumocystis jirovecii colonization can be a risk factor (17), active PJP infection, as seen in our patient, carries a significant mortality risk, especially in immunocompromised individuals. A study (18) showed that the median time from diagnosis of lung cancer to the occurrence of PJP infection was 197 days, while the interval from diagnosis to PJP infection in this renal cancer patient was 481 days. The expanding use of targeted therapy and immune checkpoint inhibitors has broadened the spectrum of patients at risk for PJP. While these agents enhance anti-tumor immunity, they may also induce immune dysregulation, prolonged T cell exhaustion, or inflammatory tissue injury—particularly in patients with pre-existing immune compromise due to advanced malignancy, malnutrition, or prior therapies. These conditions collectively create a permissive environment for Pneumocystis jirovecii reactivation or *de novo* infection (19, 20). Due to the long interval from onset to diagnosis, the optimal treatment opportunity is often missed, which ultimately affects the treatment outcome.

Since Pneumocystis jirovecii cannot be cultured *in vitro*, laboratory diagnosis is crucial for confirmation. Clinical detection methods include (1–3)-β-D-glucan serological testing, sputum culture, metagenomic next-generation sequencing (mNGS), and quantitative PCR (qcPCR) (21). Studies (22–24) have shown that (1–3)-β-D-glucan testing may have false positive results when differentiating other fungal infections. Bronchoalveolar lavage fluid (BALF) mNGS is a sensitive tool for diagnosing PJP, especially in cases where routine tests are negative but clinical suspicion is high. However, its detection cycle is relatively long (it took 6 days from symptom onset to diagnosis in this patient), which may delay treatment.

In terms of treatment, for patients with pneumonia induced after receiving immunotherapy or other cancer therapies, especially those with advanced tumors who receive immunotherapy or chemoradiotherapy and present with unexplained progressive dyspnea and hypoxemia, PJP should be immediately suspected to avoid misdiagnosis as common pulmonary infection (25). Especially when combined with severe wheezing symptoms, Pneumocystis jirovecii infection should be highly suspected, as it increases the risk of hydropneumothorax (26, 27). Studies (28–31) have shown that for patients who have received combined immunosuppressive therapy, empirical treatment should be initiated immediately once PCP is suspected. Another relevant study (30) pointed out that for non-HIV-infected patients receiving PD-1/PD-L1 inhibitor combined with CTLA-4 inhibitor therapy, prevention of PJP is particularly critical. For non-HIV-infected patients receiving immunosuppressive agents (e.g., corticosteroids) or immunomodulatory therapies such as immune checkpoint inhibitors, PJP prophylaxis should be considered, especially in those with additional risk factors such as concurrent corticosteroid use (e.g., prednisone acetate 20 mg/day or equivalent for more than 4 weeks) or other profound immunosuppression. For patients diagnosed with PJP, trimethoprim-sulfamethoxazole (TMP-SMX) is recommended as the first-line treatment, with a conventional course of 21 days. Liver function, renal function, and urine routine indicators should be monitored during treatment. While trimethoprim-sulfamethoxazole (TMP-SMX) remains the first-line treatment for PJP (30), alternative or adjunctive therapies like atovaquone may be considered in specific situations, such as intolerance to TMP-SMX or severe cases. Moreover, two multicenter studies (30, 32) showed that adjunctive glucocorticoid therapy can be used in non-HIV patients with severe PJP complicated by acute hypoxemic respiratory failure, which may improve the prognosis, but the effect is not yet clear.

### 3.3 Clinical value of immunotherapy-related biomarkers

To improve the efficacy of immunotherapy and predict adverse reactions, the identification of effective biomarkers is of great importance. Currently, the relevant biomarkers that have been discovered mainly include the following categories:

#### 3.3.1 Biomarkers for predicting therapeutic efficacy

PD-L1 expression level: As one of the most commonly used predictive indicators, high PD-L1 expression is generally associated with favorable responses to anti-PD-1/PD-L1 therapy in various cancers such as non-small cell lung cancer (NSCLC). However, it exhibits heterogeneity and may change over time, and thus cannot be used as the sole criterion for judging therapeutic efficacy (33, 34).

Tumor mutational burden (TMB): High TMB is usually associated with the ability of tumor cells to produce more neoantigens. These neoantigens can enhance the immunogenicity of tumors, thereby improving the sensitivity and responsiveness of patients to immune checkpoint inhibitor therapy (35).

T cell infiltration: The level of intratumoral CD8 + T cell infiltration is an important predictive indicator (36). For example, the SPRY1 + CD8 + exhausted T cell population in esophageal squamous cell carcinoma (ESCC) can effectively predict the efficacy of neoadjuvant PD-1 blockade and patient survival (37).

Peripheral blood indicators: The baseline expression of IFN-*γ* and IL-10 in peripheral blood can predict the response of patients with advanced melanoma to PD-1 inhibitors. During treatment for advanced hepatocellular carcinoma patients, serum albumin (ALB) and prognostic nutritional index (PNI) are positively correlated with therapeutic efficacy, while alpha-fetoprotein (AFP) and platelet-to-lymphocyte ratio (PLR) are negatively correlated (38).

Genomic and transcriptomic characteristics: Genomic and transcriptomic analysis of triple-negative breast cancer (TNBC) can classify subtypes and predict treatment responses (39). The formin protein DIAPH1 can predict the response to anti-PD-1/PD-L1 immunotherapy and is positively correlated with immune factors, thus serving as a surrogate biomarker for predicting immunotherapeutic responses (40).

#### 3.3.2 Biomarkers for predicting immune-related adverse events (irAEs)

irAEs can affect multiple organs, including pneumonitis (CIP), colitis, and endocrine diseases (41, 42). Research on relevant biomarkers is still ongoing, and susceptibility gene loci related to autoimmune diseases are considered potential predictive indicators (43). For cancer patients receiving immunotherapy, combined detection of biomarkers may help screen high-risk populations and optimize treatment regimens.

The patient in this case was a patient with advanced renal clear cell carcinoma and multiple metastases. After multiple lines of targeted combined immunotherapy, his immune function was impaired, and he developed secondary PJP infection, complicated by severe complications such as hydropneumothorax. This suggests that for high-risk patients (such as those with advanced tumors and after multiple lines of immunotherapy), biomarker monitoring should be combined with clinical symptom assessment to identify the risk of infection and irAEs at an early stage.

### 3.4 Potential mechanisms of PJP-associated Hydropneumothorax in the setting of immunotherapy

Spontaneous pneumothorax or hydropneumothorax is a rare but life-threatening complication of PJP, reported predominantly in HIV-infected individuals with extensive cystic lung destruction. In non-HIV, ICI-treated patients, the pathophysiology may differ substantially. We propose several synergistic mechanisms in this case:

(1) Necrotizing pneumonitis and subpleural cyst formation. Pneumocystis jirovecii adheres tightly to type I alveolar epithelial cells, inducing direct cytotoxicity and desquamation. In immunocompromised hosts, the organism proliferates massively, leading to foamy alveolar exudates, interstitial inflammation, and necrosis. When necrotic foci communicate with the pleural space via ruptured subpleural cysts or broncho-pleural fistulas, pneumothorax ensues. The concomitant exudative pleural effusion—driven by intense local inflammation—results in hydropneumothorax.
(2) Immune reconstitution-like phenomenon under ICIs. Unlike HIV-associated PJP, where pneumothorax typically occurs after immune recovery, ICI therapy may create a “checkpoint inhibitor-associated immune reconstitution inflammatory syndrome (IRIS)-like” state. PD-1 blockade rapidly restores effector T cell function in the lung, potentially triggering a hyperinflammatory response against residual Pneumocystis jirovecii antigens. This exaggerated Th1/Th17 response can exacerbate tissue necrosis and pleural erosion, as supported by preclinical models of PD-1 deficiency aggravating PJP immunopathology.
(3) Impaired tissue repair due to anti-angiogenic agents. The patient received sequential axitinib and sunitinib, both potent VEGF inhibitors. VEGF is critical for alveolar epithelial repair and microvascular integrity. Concurrent use of anti-angiogenic agents may delay healing of subpleural necrotic lesions, predisposing to persistent air leak and secondary pleural fluid accumulation.

Collectively, these mechanisms suggest that hydropneumothorax in ICI-treated PJP patients is not merely a bystander event but a reflection of accelerated immunopathology superimposed on impaired structural repair. This underscores the need for early thoracic imaging and low-threshold mNGS testing in ICI recipients with unexplained respiratory deterioration.

## 4 Conclusion

Patients with left renal clear cell carcinoma treated with immune checkpoint inhibitors (ICIs) can develop Pneumocystis jirovecii pneumonia (PJP) complicated by left-sided hydropneumothorax, presenting with progressive dyspnea, shortness of breath, and hypoxemia. Although anti-PD-1 inhibitors can block the PD-1/PD-L1 pathway and enhance anti-tumor T cell effects, they may also disrupt the body’s immune balance against Pneumocystis. Although anti-PD-1 inhibitors are immunostimulatory rather than directly immunosuppressive, they may disrupt pulmonary immune tolerance and, in the setting of advanced cancer-related immune frailty, cumulative treatment burden, and nutritional depletion, collectively increase susceptibility to PJP. This case underscores that PJP risk in ICI-treated patients should be assessed through a composite of clinical factors, not merely by drug class. We hypothesize that Pneumocystis-induced pulmonary tissue necrosis and subsequent pleural structural destruction were the key pathological mechanisms contributing to the development of hydropneumothorax in this patient. In this case, the pneumothorax masked the imaging features of PJP, and the delay in etiological testing led to a delay in diagnosis, accompanied by severe complications such as sepsis and respiratory failure, resulting in a poor prognosis. Clinicians should be highly vigilant when cancer patients receiving ICI treatment present with dyspnea and hypoxemia, and include PJP complicated by pneumothorax/hydropneumothorax in the differential diagnosis. It is necessary to make full use of biomarkers to optimize treatment strategies, achieve rapid identification and timely intervention of high-risk patients, and reduce the mortality rate of PJP. We hypothesize that Pneumocystis-induced pulmonary tissue necrosis and subsequent pleural structural destruction were the key pathological mechanisms contributing to the development of hydropneumothorax in this patient.

## Data Availability

The original contributions presented in the study are included in the article/supplementary material, further inquiries can be directed to the corresponding author.
