# Correction: Case Report: A rare case of Pneumocystis jirovecii infection with left hydropneumothorax following immunotherapy for stage IVB clear cell renal cell carcinoma

**DOI:** 10.3389/fmed.2026.1838047

**Published:** 2026-04-01

**Authors:** Frontiers Production Office

**Affiliations:** Frontiers Media SA, Lausanne, Switzerland

**Keywords:** hydropneumothorax, immune checkpoint inhibitors, immunotherapy, PJP, renal carcinoma

There was a mistake in Table 1 as published. The cells in the bottom row were incorrectly formatted. The corrected Table 1 appears below.

**Table 1:** Metagenomic next-generation sequencing (mNGS) of pathogenic microorganisms in bronchoalveolar lavage fluid.

				Complex/species		
Type	Name	Sequence count	Relative abundance	Name	Sequence count	Coverage
Fungal list						
Fun	Pneumocystis	1	4.0%	*Pneumocystis jirovecii*	1	0.0006%
DNA/RNA virus list						
DNA	Alphatorquevirus	903	95.053%	*Torque teno virus 20*	774	90.1051%
List of suspected colonizing and/or background microorganisms						
G+	Cutibacterium	6	–	*Cutibacterium acnes*	5	–

The references Zhou et al. (2023) and Raimondi et al. (2020) were erroneously omitted from the reference list. These have now been added as reference 31 and 33. The references appear below.

31. Zhou S, Aitken SL. Prophylaxis against *Pneumocystis jirovecii* pneumonia in adults. *JAMA*. (2023) 330:182–3. doi: 10.1001/jama.2023.9844

33. Raimondi A, Sepe P, Zattarin E, Mennitto A, Stellato M, Claps M, et al. Predictive biomarkers of response to immunotherapy in metastatic renal cell cancer. *Front Oncol*. (2020) 10:1644. doi: 10.3389/fonc.2020.01644

References 31–43 have been re-numbered accordingly, and appear below:

31. Zhou S, Aitken SL. Prophylaxis against *Pneumocystis jirovecii* pneumonia in adults. *JAMA*. (2023) 330:182–3. doi: 10.1001/jama.2023.9844

32. Kamel T, Baudry T, Le Fouler L, Reignier J, Bouadma L, Frat JP, et al. Pneumocystis pneumonia in intensive care: clinical spectrum, prophylaxis patterns, antibiotic treatment delay impact, and role of corticosteroids. A French multicentre prospective cohort study. *Intensive Care Med*. (2024) 50:1228–39. doi: 10.1007/s00134-024-07489-2

33. Raimondi A, Sepe P, Zattarin E, Mennitto A, Stellato M, Claps M, et al. Predictive biomarkers of response to immunotherapy in metastatic renal cell cancer. *Front Oncol*. (2020) 10:1644. doi: 10.3389/fonc.2020.01644

34. Kono K, Nakajima S, Mimura K. Current status of immune checkpoint inhibitors for gastric cancer. *Gastric Cancer*. (2020) 23:565–78. doi: 10.1007/s10120-020-01090-4

35. Lei Q, Wang D, Sun K, Wang L, Zhang Y. Resistance mechanisms of anti-PD1/PDL1 therapy in solid tumors. *Front Cell Dev Biol*. (2020) 8:672. doi: 10.3389/fcell.2020.00672

36. Yang M, Yang H, Ji Y, Lu Y, Zhou Y, Zhang H, et al Mechanisms, combination therapy, and biomarkers in cancer immunotherapy resistance. *Cell Commun Signal*. (2024) 22:338. doi: 10.1186/s12964-024-01711-w

37. Liu Z, Zhang W, Wu J, Li D, Mao Y, Zhu Y, et al. Progenitor-like exhausted SPRY1+CD8+ T cells potentiate responsiveness to neoadjuvant PD-1 blockade in esophageal squamous cell carcinoma. *Cancer Cell*. (2023) 41:1852–70. doi: 10.1016/j.ccell.2023.09.011

38. Jiang Y, Zhang C, Cao Y, Wang K. Nutrition and metabolism status alteration in advanced hepatocellular carcinoma patients treated with anti-PD-1 immunotherapy. *Support Care Cancer*. (2020) 28:5569–79. doi: 10.1007/s00520-020-05478-x

39. Jiang YZ, Ma D, Suo C, Shi J, Xue M, Hu X, et al. Genomic and transcriptomic landscape of triple-negative breast cancers: subtypes and treatment strategies. *Cancer Cell*. (2019) 35:428–440.e5. doi: 10.1016/j.ccell.2019.02.001

40. Mei J, Hu H, Li Y, Lv S, Cheng Y, Liu Y, et al. Formin protein DIAPH1 positively regulates PD-L1 expression and predicts the therapeutic response to anti-PD-1/PD-L1 immu-notherapy. *Clin Immunol*. (2023) 246:109204. doi: 10.1016/j.clim.2022.109204

41. Moda M, Hata Y, Takamori R, Hirai K, Morita M, Ikeda T, et al. Tumor invasion in the central airway is a risk factor for early-onset checkpoint inhibitor pneumonitis in patients with non-small cell lung cancer. *Thorac Cancer*. (2020) 11:3576–84. doi: 10.1111/1759-7714.13703

42. Dudzińska M, Szczyrek M, Rola R, Krawczyk P, Milanowski J. Endocrine adverse events of Nivolumab in non-small cell lung Cancer patients-literature review. *Cancers*. (2020) 12:2314. doi: 10.3390/cancers12082314

43. Hoefsmit EP, Rozeman EA, Haanen J, Blank CU. Susceptible loci associated with autoimmune disease as potential biomarkers for checkpoint inhibitor-induced immune-related adverse events. *ESMO Open*. (2019) 4:e000472. doi: 10.1136/esmoopen-2018-000472

The in-text citations for references 42 and 43 were missed in the article and were therefore not cited. The citations have now been inserted in section **3 Discussion**, *3.3.2 Biomarkers for predicting immune-related adverse events (irAEs)*, Paragraph 1. The paragraph has been corrected to:

“irAEs can affect multiple organs, including pneumonitis (CIP), colitis, and endocrine diseases (41, 42). Research on relevant biomarkers is still ongoing, and susceptibility gene loci related to autoimmune diseases are considered potential predictive indicators (43). For cancer patients receiving immunotherapy, combined detection of biomarkers may help screen high-risk populations and optimize treatment regimens.”

The original version of this article has been updated.

